# The predictability of graft thickness for Descemet’s stripping automated endothelial keratoplasty using a mechanical microkeratome system

**DOI:** 10.1038/s41598-022-26679-w

**Published:** 2022-12-23

**Authors:** Sota Nishisako, Takefumi Yamaguchi, Yuki Kusano, Kazunari Higa, Dai Aoki, Chiaki Sasaki, Jun Shimazaki

**Affiliations:** 1 Cornea Center and Eye Bank, Tokyo Dental College, Ichikawa General Hospital, Chiba, Japan; 2 Department of Ophthalmology, Tokyo Dental College, Ichikawa General Hospital, Chiba, Japan; 3 Department of Ophthalmology, Kumamoto University, Kumamoto, Japan

**Keywords:** Medical research, Risk factors

## Abstract

Descemet's stripping automated endothelial keratoplasty (DSAEK) is used for treating corneal endothelial dysfunction, and the postoperative visual acuity outcome depends on the thickness of the graft. We created a simple nomogram using factors affecting the cutting thickness during graft preparation via a mechanical microkeratome system for DSAEK. This retrospective study was conducted from May 2018 through October 2022 and included donor eyes cut by automatic methods. We measured the graft thickness, cutting accuracy, and assessed ten variables with donor/cornea-related factors potentially affecting the cutting thickness. Subsequently, we created a simple nomogram. We analyzed 81 donor tissues, and the donor median age was 76 years. The mean central graft thickness was 122.2 μm, with 62% of the grafts that could be cut within the target central graft thickness range. Comparatively, donor corneas from those with cardiac diseases were cut deeper (*P* = 0.007). The developed nomogram provided a 83% probability of estimating the post-cutting graft thickness within 25 µm. Our nomogram, which considers cause of death, enables reproducible production of graft of a desired thickness. A detailed analysis of donor tissues, including the cause of donor death and the characteristics from pressurization to cutting, will enable more precise DSAEK graft preparation.

## Introduction

Descemet's stripping automated endothelial keratoplasty (DSAEK) is widely used for treating corneal endothelial dysfunction^1,2^. A high-quality pre-cut donor tissue is necessary for a successful surgery and for achieving good visual acuity^3–5^. Several factors influence good outcomes following DSAEK; of these, the thickness of the graft affects the postoperative visual acuity^2,3^. A protocol to achieve the desired graft thickness without perforation should be developed to improve patient prognosis and to conserve donor corneas due to a shortage worldwide^6^.

DSAEK tissue is most often processed by a microkeratome and the cutting depth is adjusted to control the thickness of the resulting posterior lenticule^7^. Manual microkeratome cutting produces grafts of variable thickness because of the difficulty in maintaining constant conditions, and its results are less reproducible^8,9^. Recently, some operators have prepared DSAEK grafts using a mechanical microkeratome system, in which a pass is automatically performed by the instrument, while a constant artificial chamber internal pressure (IP) is maintained^2,10,11^. Thus, we hypothesized that it was possible to identify other factors affecting the cutting thickness. We then developed an accurate nomogram to create the desired thickness grafts under fixed cutting conditions using a mechanical microkeratome system.

We sought to create a simple nomogram using each microkeratome head size by examining donor/cornea-related factors affecting the cutting thickness of DSAEK graft preparation under fixed conditions of IP settings and cutting speed by a mechanical microkeratome system.

## Results

### Baseline characteristics of donor corneas

We prepared 89 donor tissues using the mechanical microkeratome system for DSAEK during the study. All cut donor corneas showed no perforation or other complications and were used for DSAEK. Table 1 summarizes the baseline donor/corneal demographics. Eighty-one randomly selected donor corneas were analyzed, the age ranged from 28 to 95 years (median, 76 years), and 54 (67%) were men. Causes of death included cancer, cardiac disease, cerebrovascular accident, and others in 21 (26%), 24 (30%), 17 (21%), and 19 (23%) donors, respectively. The mean endothelial cell density (ECD) was 2639 cells/mm^2^ (range 2053–3175 cells/mm^2^); eight grafts (10%) displayed good epithelial status before cutting, whereas 73 grafts displayed non-good status (defect, exposure, and rough).

**Table 1:** Baseline donor and corneal demographics.

Donor and corneal characteristics	
The number of eyes	81
Donor age, median (IQR), range, y	76 (61–86), 28–95
Donor sex, man, *n* (%)	54 (67)
History of diabetes mellitus, *n* (%)	11 (14)
**Donor lens status, *n* (%)**	
Phakic	68 (84)
IOL	13 (16)
**The cause of death, *n* (%)**	
Cancer	21 (26)
Cardiac disease	24 (30)
Cerebrovascular accident	17 (21)
Others	19 (23)
D–P Time, median (IQR), range, h	6.8 (5.6–9.3), 1.1–25.4
D–C Time, mean (SD), range, d	4.9 (1.3), 2–8
Graft ECD, mean (SD), range, cells/mm^2^	2639 (259.0), 2053–3175
**Epithelial status, *n* (%)**	
Good	8 (10)
Non-good	73 (90)
**Endothelial folds, *n* (%)**	
None	18 (22)
Mild to moderate	63 (78)

D–C, death to cutting; D–P, death to preservation; ECD, endothelial cell density; IOL, intraocular lens; IQR, interquartile range; SD, standard deviation.

### Corneal thickness profiles

**Figure 1:**
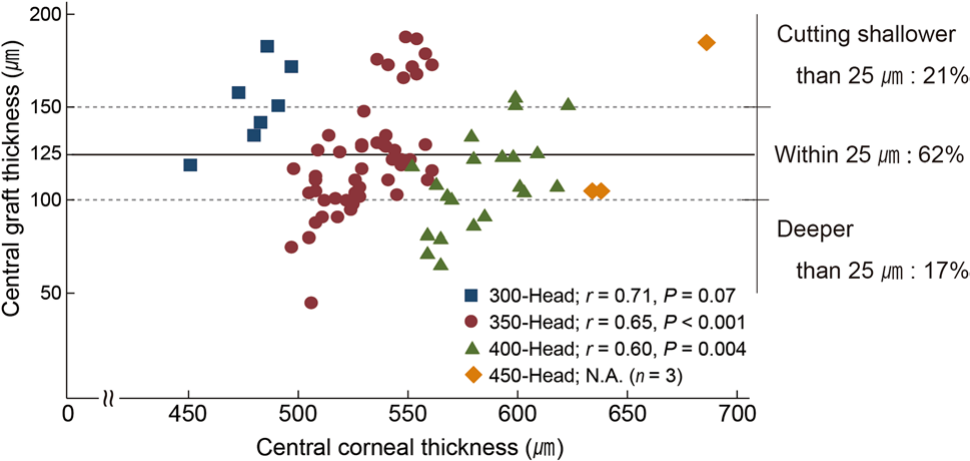
A scatter plot of the central donor corneal thickness versus post-cut central graft thickness by each microkeratome head size. The central donor corneal thickness and post-cut central graft thickness are positively correlated (Pearson *r* = 0.60 to 0.65; *P* < 0.01) in 350- and 400-microkeratome head sizes. Approximately 62% of the grafts could be cut within the target central graft thickness range (125 ± 25 μm); 21% of the grafts were cut to a depth shallower than 25 μm, and 17% were cut deeper. The solid line represents the target thickness (125 μm); the dashed line indicates the ± 25 μm range; and the square indicates the 300-, 350- (circle), 400- (triangle), and 450-heads (diamond).

Table 2 summarizes the thickness profiles sorted by microkeratome head sizes. Total donor corneas were cut within 45 μm to 188 μm (mean 122.2 μm), and the mean cutting depth was greater depending on the change in the microkeratome head size (from 300- to 450-head). Figure 1 depicts a scatter plot of the central donor corneal thickness versus post-cut central graft thickness by each microkeratome head size. The central donor corneal thickness was positively correlated with the post-cut central graft thickness (Pearson *r* = 0.60 to 0.65, *P* < 0.01) in 350- and 400-microkeratome head sizes. Approximately 62% of the grafts could be cut within the target central graft thickness range (125 ± 25 μm); 21% of the grafts were cut to a depth shallower than 25 μm, and 17% were cut deeper.

**Table 2:** Thickness profile by the microkeratome head size.

	Thickness profile
**Total**	
Eyes, N	81
Corneal thickness, mean (SD), range, μm	544.8 (41.0), 451–686
Graft thickness, mean (SD), range, μm	122.2 (30.9), 45–188
**300-head subgroup**	
Eyes, *n,* (%)	7 (8)
Corneal thickness, mean (SD), range, μm	480.1 (15.0), 451–497
Cutting depth, mean (SD), range, μm	328.7 (15.4), 303–345
Graft thickness, mean (SD), range, μm	151.4 (21.9), 119–183
**350-head subgroup**	
Eyes, *n,* (%)	50 (62)
Corneal thickness, mean (SD), range, μm	530.9 (18.4), 497–561
Cutting depth, mean (SD), range, μm	408.1 (23.4), 360–461
Graft thickness, mean (SD), range, μm	122.8 (30.7), 45–188
**400-head subgroup**	
Eyes, *n,* (%)	21 (26)
Corneal thickness, mean (SD), range, μm	584.2 (20.8), 552–623
Cutting depth, mean (SD), range, μm	474.3 (21.3), 434–511
Graft thickness, mean (SD), range, μm	109.7 (25.7), 65–155
**450-head subgroup**	
Eyes, *n,* (%)	3 (4)
Corneal thickness, mean (SD), range, μm	652.7 (28.9), 634–686
Cutting depth, mean (SD), range, μm	521.0 (17.4), 501–533
Graft thickness, mean (SD), range, μm	131.7 (46.2), 105–185

SD, standard deviation.

### Nomogram for artificial chamber pressurizer (ACP) and one use-plus automated (OUP-A) cutting

In the donor corneas cut by ACP/OUP-A, the cause of death (*P* < 0.001) was identified as a potential factor affecting the cutting thickness of the ten variables with donor/cornea-related factors tested in univariate analysis (Table 3). From the results of the factors associated with cutting depth, we structured a linear mixed-effect model for estimating the post-cut graft thickness on the central donor corneal thickness, microkeratome head size (fixed effects), and the cause of death (random effect). In model selection, we selected death from a cardiac disease as a useful parameter for deeper cutting (*P* = 0.007). Donor corneas from those with cardiac diseases were estimated to cut significantly deeper (20.66 μm) than those with other causes of death. Eventually, the selected model was calculated as follows:

**Table 3:** Factors associated with deeper cutting (N = 81).

Prognostic factor	*n*	Univariate models^a^		Linear mixed-effect models^b^
		*n* (%)	*p*-value	*p*-value
**Donor age, y**				
28–75	38	19 (50)	0.25	
76–94	43	27 (63)		
**Donor sex**				
Man	54	28 (52)	0.24	
Woman	27	18 (67)		
**History of diabetes mellitus**				
No	70	39 (56)	0.75	
Yes	11	7 (64)		
**Donor lens status**				
Phakic	68	38 (57)	0.77	
IOL	13	8 (62)		
**The cause of death**				
Cancer	21	12 (57)	< 0.001	0.99
Cardiac disease	24	20 (83)		0.007
Cerebrovascular accident	17	2 (12)		0.10
Others	19	12 (63)		0.84
**D-P time, h**				
1.1–6.7	39	21 (54)	0.61	
6.8–25.4	42	25 (60)		
**D-C time, d**				
3.0–4.9	32	19 (59)	0.70	
5.0–8.0	49	27 (55)		
**Graft ECD, cells/mm**^**2**^				
2053–2639	40	24 (60)	0.57	
2640–3175	41	22 (54)		
**Epithelial status**				
Good	8	3 (38)	0.28	
Non-good	73	43 (59)		
**Endothelial folds**				
None	18	9 (50)	0.59	
Mild to moderate	63	37 (59)		

D–C, death to cutting; D–P, death to preservation; ECD, endothelial cell density; IOL, intraocular lens.

^a^The percentage of donor corneas that were cut deeper than the mean value in each microkeratome head size by the chi-square test/Fisher’s exact test.

^b^Analyzed with a linear mixed-effect model adjusted for the corneal thickness and microkeratome head size (fixed effect), and the cause of death (random effect).

Model 1: death from non-cardiac disease, microkeratome 300-head size;

$$GT_{\text{non-cardiac-300}} = 0.9972 \times CT - 318.54$$

Model 2: death from non-cardiac disease, microkeratome 350-head size;

$$GT_{\text{non-cardiac-350}} = 0.9972 \times CT - 402.47$$

Model 3: death from non-cardiac disease, microkeratome 400-head size;

$$GT_{\text{non-cardiac-400}} = 0.9972 \times CT - 462.10$$

Model 4: death from non-cardiac disease, microkeratome 450-head size;

$$GT_{\text{non-cardiac-450}} = 0.{9972 } \times CT - 519.20$$

Model 5: death from cardiac disease, microkeratome 300-head size;

$$GT_{\text{cardiac-300}} = 0.9972 \times CT - 318.54-{ 2}0.{66}$$

Model 6: death from cardiac disease, microkeratome 350-head size;

$$GT_{\text{cardiac-350}} = 0.9972 \times CT - 402.47 - 20.66$$

Model 7: death from cardiac disease, microkeratome 400-head size;

$$GT_{\text{cardiac-400}} = 0.9972 \times CT - 462.10 - { 2}0.{66}$$

Model 8: death from cardiac disease, microkeratome 450-head size;

$$GT_{\text{cardiac-450}} = 0.{9972 } \times CT - 519.20 - 20.66$$

where: *GT* = central graft thickness, μm, *CT* = central corneal thickness, μm.

**Figure 2:**
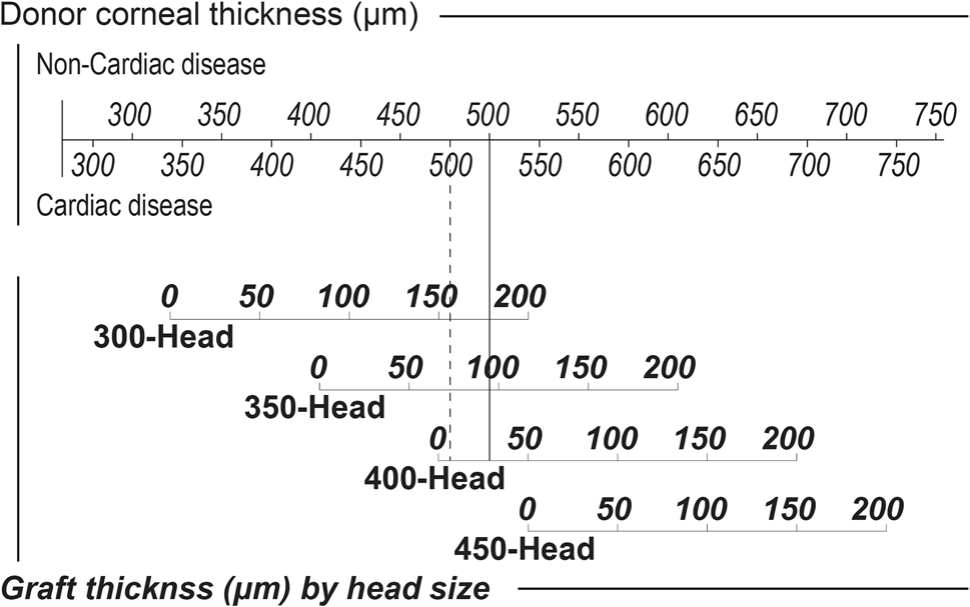
A nomogram to predict the post-cut central graft thickness for a mechanical microkeratome cutting with an artificial chamber pressurizer and one use-plus automated. For example, the central graft thickness of a 500-μm cornea from a donor with non-cardiac disease (solid line) is predicted to be 180.1 μm, 96.1 μm, and 36.5 μm for a cut using 300-, 350-, and 400-head sizes, respectively. The thickness for donors with cardiac disease as the cause of death (dashed line) is predicted to be 159.4 μm, 75.5 μm, and 15.8 μm for a cut using 300-, 350-, and 400-head sizes, respectively.

**Figure 3:**
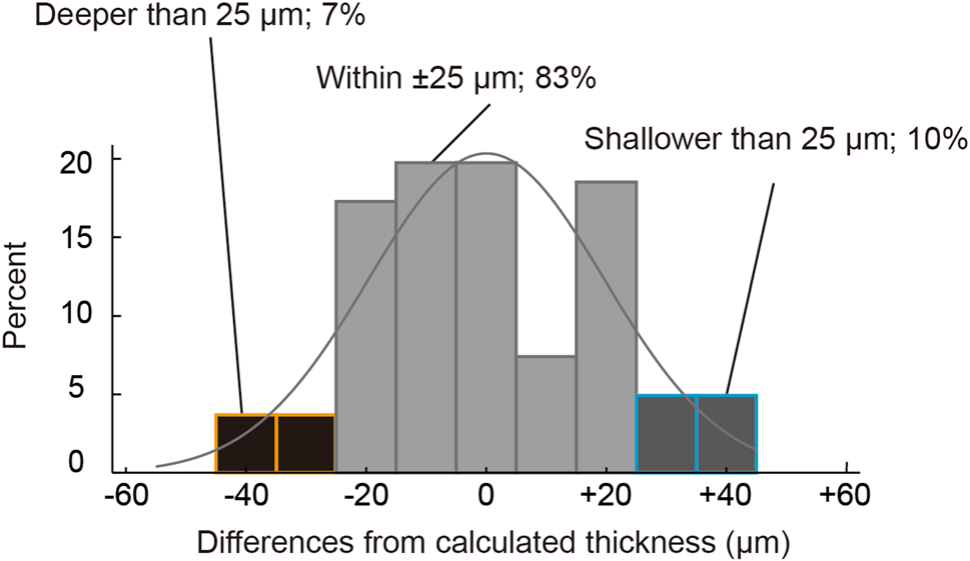
Cutting predictability of the nomogram. This nomogram has estimated the post-cut central graft thickness within 25 μm with a 83% probability. It poses an 7% risk of cutting 25 μm deeper than that predicted. The solid line represents a normal estimate.

Based on the models, Fig. 2 depicts a nomogram to predict the post-cut central graft thickness from the central donor corneal thickness for each microkeratome head size and cause of death (cardiac/non-cardiac disease). Figure 3 depicts the accuracy of the developed nomogram. The nomogram estimated the post-cut central graft thickness within 25 μm with a theoretical 83% probability, and testing with corneas that were not used for modeling provided a 75% (6/8) probability.

## Discussion

Optimizing head selection of the microkeratome and minimizing the error range between the dissected graft thickness and target thickness are the most important factors for obtaining the desired graft thickness in post-cutting for DSAEK. We identified the cause of donor death as a factor that resulted in variations in the cut thickness. Moreover, we developed a DSAEK cutting nomogram based on the donor with cardiac/non-cardiac diseases by the microkeratome head size.

The average DSAEK graft thickness prepared by ACP/OUP-A at Cornea Center and Eye Bank (CCEB, Chiba, Japan) was 122.2 μm. In addition, 350-head was the most frequently selected size (Table 2). Despite the correlation between the post-cut central graft thickness and the central donor corneal thickness, we identified variations in the post-cut central graft thickness even within the identical central donor corneal thickness range. The cutting speed^12,13^ and IP settings^10,14^ affect the thickness of the cut. In CCEB, DSAEK grafts cut by ACP/OUP-A displayed a narrower thickness range and were cut with higher accuracy than grafts from conventional manual cutting (Supplemental Fig. 1). However, the probability of obtaining grafts within a range of ± 25 μm from the target was approximately 60%, notwithstanding constant cutting conditions (Fig. 1). Clerici et al. examined the reproducibility of graft thickness by the pressurization method for preparing ultrathin-DSAEK (i.e., the target central graft thickness was under 100 μm) and reported on error ranges for the dissected graft thickness^11^, as in the present study. This indicates the existence of factors other than the cutting operation that affect the cutting error. In other words, operators should consider other factors, despite using only the central donor corneal thickness as an indicator of pre-cut.

We identified the cause of death as a factor affecting the cutting depth in the preparation for DSAEK graft under fixed conditions. Predicting graft thickness after cutting with a model adjusted for donor cause of death provided about a 20% (62% to 83%) improvement in the theoretical probability of a target range compared to predicting with only donor corneal thickness (Figs. 1, 3). There have been reports on the association of patients’ intraocular pressure and corneal biomechanical characteristics (e.g., corneal hysteresis/corneal resistance factor) with systemic and lifestyle factors, including age, sex, systemic blood pressure, height, smoking, alcohol use, diabetes, glycosylated hemoglobin level, and systemic lupus erythematosus^15–17^. Furthermore, Suzuki et al. reported that cardiac abnormalities (e.g., atrial fibrillation, left ventricular hypertrophy, and bradycardia) were associated with the development and severity of glaucoma^18^. Considering that the central donor corneal thickness before cutting did not differ for each cause of donor death (*P* = 0.33, one-way analysis of variance, data not shown) and constant IP was maintained by ACP at 200 mmHg, the affecting cutting depth can be attributed to the tissue resistance to deformation characteristics of the donor cornea (e.g., bounce and stiffness) during pressurization or cutting. Corneas from donors due to cardiac death are considered to be hard in consistency and low in resilience. Our data may indicate that, during procurement of a cornea from a cardiac-death donor using a microkeratome, the angle at the blade entry point is deeper, resulting in a deeper cut to the tissue. The cause of death may reflect the condition of the cornea rather than an independent biomarker. Further detailed data/analysis are needed because potentially include factors not addressed in this study as confounding factors. Donor corneas used for cutting are processed onto the sclerocorneal button, thus making it difficult to determine their biomechanical characteristics. Therefore, changes in the shape in response to the IP have not been studied. The measurement and understanding of the shape of tissue resistance to deformation characteristics of the donor cornea under IP with instruments, such as the Ocular Response Analyzer^19^ will further improve the cutting accuracy in the future.

A nomogram consisting only of the central donor corneal thickness and microkeratome head size would have limitations in predicting the post-cut central graft thickness. We constructed a model that predicted the graft thickness after cutting based on the central donor corneal thickness, microkeratome head size, and the cause of death. In addition, we illustrated the results as a nomogram (Fig. 2). In this model, providing 83% of the predictions made on the test data set were within the target graft thickness. For example, in cutting donor cornea with a 500-μm central thickness, the central graft thickness was predicted to be 180.1 μm, 96.1 μm, and 36.5 μm for cuts using 300-, 350-, and 400-heads, respectively, for donors with a non-cardiac disease. By contrast, it was predicted to be 159.4 μm, 75.5 μm, and 15.8 μm for cuts using 300-, 350-, and 400-heads, respectively, for donors with cardiac disease as the cause of death. Bae et al. reported on a nomogram to predict the thickness of DSAEK corneal grafts by linear regression models, including the donor corneal thickness and donor age, using larger sample sizes (768 corneal data)^20^. The developed nomogram indicated that the older the donor, the deeper the cut. Moreover, our findings revealed a tendency for older donors to have deeper cuts; nonetheless, there was no statistically significant difference in the depth of cut by donor age. The age-adjusted nomogram could predict the thickness to be within 25 μm 80% of the time, like the accuracy level of our nomogram that adjusted for the cause of death (Fig. 3). Some reports have included epithelial removal in the nomogram^21^, which may be related to the thickness adjustment of the epithelial portion of the cornea. In the present study, we did not extract the condition of the donor cornea epithelium as a factor affecting the depth of cutting.

Investigators have developed various cutting techniques, such as the double-pass method^22,23^, slow pass technique^12,13^, and drying method^24,25^, to obtain thinner DSAEK grafts. However, the simplest or easiest way to obtain thin grafts involves selecting a microkeratome head size that cuts deeper. Microkeratome head selection for obtaining thinner grafts (i.e., Ultra-thin DSAEK grafts) may also be possible in our developed nomogram. However, considering that our nomogram had an 7% risk of cutting 25 μm deeper and a maximum difference of cutting 44.0 μm deeper than that predicted value (Fig. 3), setting the target graft thickness < 50 μm, termed the nano-thin DSAEK^26^, is considered risky for perforation.

This study had some limitations. First, all the donors were Japanese, and their corneas were preserved in a cold-storage corneal medium at a low temperature. Researchers should perform similar studies and comparisons in other regions of the world. This is because the shape of corneoscleral tissues and/or corneal biomechanical characteristics may depend on the race and preservation method (i.e., organ culture preservation). Second, there is no standard method for measuring the thickness of the donor cornea. Several institutions measure the corneal thickness before/after cutting with a pachymeter, and not anterior segment optical coherence tomography. An accurate installation of the developed nomogram warrants understanding differences between the measurement devices. Third, our sample size was relatively small. An accurate modeling requires the consideration of several variables and a larger sample size.

In conclusion, the cause of death (i.e., cardiac disease) affected the cutting depth under fixed settings with a mechanical microkeratome system. It is possible to achieve 83% probability of producing grafts within ± 25 m of the target thickness using the nomogram that predicted the post-cut central graft thickness from the central donor corneal thickness for each microkeratome head size and cause of death (cardiac/non-cardiac disease). Furthermore, a detailed analysis of donor tissues, including the cause of donor death and characteristics from pressurization to cutting will enable an accurate DSAEK graft preparation. This, in turn, would likely optimize the use of limited donor resources and improve patient outcomes.

## Methods

### Human corneas

This single-center retrospective study was conducted from May 2018 through October 2022, with donor eyes cut by the mechanical microkeratome system at CCEB, Chiba, Japan. All donor corneal tissues were of domestic origin and donated to CCEB, preserved in a viewing storage chamber, and retained in a cold-storage corneal preservation medium (Optisol-GS solution; Bausch and Lomb Surgical, Rochester, NY, USA). All eligible donor corneas prepared for DSAEK met the medical standards of CCEB. This study was conducted according to the tenets of the Declaration of Helsinki and was approved by the Institutional Ethics Reviewer Board of the Tokyo Dental College Ichikawa General Hospital (Acceptance No. I 22-07). The need to obtain informed consent directly was waived; however, study participants were able to decline participation using an opt-out method. All data were anonymized before access and/or analysis.

### Tissue preparation

All donor tissues were stored at 4 °C and returned to room temperature before the microkeratome cut. The tissues were dissected by the mechanical microkeratome system using an artificial chamber pressurizer (ACP, Moria, Antony, France) and one use-plus automated (OUP-A, Moria)^10^. Briefly, for the ACP/OUP-A automatic protocol, we set the initial IP at 200 mmHg and constantly maintained it with ACP during the microkeratome cut. The target central graft thickness was 125 ± 25 μm. We selected the microkeratome head size within 300-, 350-, 400-, and 450-head sizes based on the central donor cornea thickness aiming for a target central graft thickness. Typically, we adapted the 300-, 350-, 400-, and 450-head in case of central donor corneal thickness < 499 μm, 500 μm–549 μm; 550 μm–599 μm; and > 600 μm, respectively. OUP-A was set on an artificial chamber and the pass was automatically performed by the instrument, with a forwarding speed of 3.0 mm/s by selecting a “speed 2” on the Evolution 3E control unit (Moria). An experienced technician (SN) performed all cutting using a single-pass technique in a laminar flow cabinet with a new disposable blade for each donor. Following cutting, the separated anterior lamella cap was replaced onto the cornea and returned to the viewing chamber filled with the cold-storage corneal preservation medium. We measured the central corneal thickness before and after the microkeratome cut using anterior segment optical coherence tomography (CASIA2; TOMEY, Nagoya, Japan)^27^. Each corneoscleral donor tissue was scanned through the transparent window of the corneal viewing chamber, without removing them from the storage medium. The chamber was held on the chinrest by a custom-built attachment, with the mounted cornea facing the image capture lens. Images were obtained along two meridians (horizontal and vertical) in manual mode. The distances from the endothelial surface to the top epithelial surface and the top of the cutting line was measured to determine the center point of each corneal image. The corneal center and graft thicknesses were measured at this center point and the mean values were subsequently calculated. This two-dimensional analysis was performed in manual mode using the scale in the image analysis software. We calculated the cutting depth by subtracting the thickness before and after the cut at the center. All tissues were evaluated for ECD and transplant suitability (i.e., epithelial/stromal/endothelial cells/endothelial folds/cutting issue) using slit-lamp microscopy performed by ophthalmologists before DSAEK.

### Data analysis

We tabulated the central donor corneal thickness and central graft thickness by the microkeratome head size and created scatter plots. We calculated the cutting accuracy as the percentage of corneas that could be cut within the target thickness range or those cut in other ranges (deeper/shallower cutting). We selected the following ten donor/donor cornea-related factors^28,29^ that could potentially affect the cutting thickness: age, sex, a history of diabetes mellitus, lens status (phakic/intraocular lens [IOL]), cause of death (cancer/cardiac disease/cerebrovascular accident/other diseases), time from death until preservation, time from death until cutting, ECD, graft epithelial status (good/non-good), and graft endothelial folds (none/mild/moderate). Using the extracted factors, we constructed a model to predict the post-cut graft thickness from the donor corneal thickness and microkeratome head size to create a simple nomogram. A cut graft thickness model was created by randomly selecting 81 eyes, and the remaining 8 eyes were used to evaluate the accuracy of the created model.

### Statistical analyses

The effect size estimates were calculated using pilot cut data (*n* = 50) since the variation in graft thickness was dependent on the cutting method^10,29^. An effect size of 0.61 (Cohen’s *d*) was estimated to detect a difference of more than 20 μm in the thickness of the cut graft by donor/donor cornea-related factors. In the linear mixed-effects model with two clusters, a sample size of approximately 81 observations was sufficient to achieve 80% power to detect effect size of Cohen’s *d* = 0.61 at an alpha level of 0.05. We assessed the normality of data using the Shapiro–Wilk test and a normal Q-Q plot. We estimated the Pearson's correlation coefficient for the relationship between the central donor corneal thickness and central post-cut graft thickness. Potential donor/donor cornea-related factors affecting the cutting depth were identified based on a univariate analysis with the outcome of cutting depth being deeper or shallower than the mean thickness (*P* < 0.05). In this analysis, the collected categorical data were transformed into a dummy, and continuous variables were dichotomized with the mean/median for use as categorical data. We used a linear mixed-effect model to predict the post-cut graft thickness^30^, with the central donor corneal thickness and microkeratome head size (fixed effect) adjusted for potential donor/donor cornea-related factors (random intercepts) that affected the cutting depth. The variance estimation method was restricted maximum likelihood, and the regression model was selected using Akaike's information criterion with a *P* < 0.05 as the variable selection criterion. Statistical analyses for cutting accuracy were conducted using STATA/IC 16.0 for Windows (StataCorp LP, College Station, TX). We used R version 4.2.1 for Windows (R Foundation for Statistical Computing, Vienna, Austria) for the sample size calculation in a linear mixed-effect model (sjstats package) and for thickness modeling (lme4 package). All reported *P*-values were two-sided, and values < 0.05 were considered statistically significant.

## Supplementary Information

Supplementary Figure 1.

## Data Availability

The data that used and/or analyzed during the current study are available from the corresponding author upon reasonable request.

## References

[1] Takahashi A (2021). Trends in surgical procedures and indications for corneal transplantation over 27 years in a tertiary hospital in Japan. Jpn. J. Ophthalmol..

[2] Yeu E (2021). Posterior lamellar keratoplasty: Techniques, outcomes, and recent advances. J. Cataract Refract. Surg..

[3] Dickman MM (2016). A randomized multicenter clinical trial of ultrathin Descemet stripping automated endothelial keratoplasty (DSAEK) versus DSAEK. Ophthalmology.

[4] Terry MA (2018). Donor, recipient, and operative factors associated with graft success in the cornea Preservation Time Study. Ophthalmology.

[5] Nishisako S (2022). Donor-related risk factors for graft decompensation following Descemet's stripping automated endothelial keratoplasty. Front. Med..

[6] Gain P (2016). Global survey of corneal transplantation and eye banking. JAMA Ophthalmol..

[7] Woodward MA, Titus M, Mavin K, Shtein RM (2012). Corneal donor tissue preparation for endothelial keratoplasty. J. Vis. Exp..

[8] Price MO (2008). Central thickness variation in precut DSAEK donor grafts. J. Cataract Refract. Surg..

[9] Rose L, Briceño CA, Stark WJ, Gloria DG, Jun AS (2008). Assessment of eye bank-prepared posterior lamellar corneal tissue for endothelial keratoplasty. Ophthalmology.

[10] Nishisako S, Aoki D, Sasaki C, Higa K, Shimazaki J (2018). Comparison of artificial anterior chamber internal pressures and cutting systems for Descemet's stripping automated endothelial keratoplasty. Transl. Vis. Sci. Technol..

[11] Clerici R (2021). Single-pass mikrokeratome and anterior chamber pressurizer for the ultrathin descemet-stripping automated endothelial keratoplasty graft preparation. Cornea.

[12] Bhogal MS, Allan BD (2012). Graft profile and thickness as a function of cut transition speed in Descemet-stripping automated endothelial keratoplasty. J. Cataract Refract. Surg..

[13] Vajpayee RB, Maharana PK, Jain S, Sharma N, Jhanji V (2014). Thin lenticule descemet's stripping automated endothelial keratoplasty: Single, slow pass technique. Clin. Exp. Ophthalmol..

[14] Romano V (2015). Reliability of the effect of artificial anterior chamber pressure and corneal drying on corneal graft thickness. Cornea.

[15] Foster PJ (2011). Intraocular pressure and corneal biomechanics in an adult British population: The EPIC-Norfolk eye study. Investig. Ophthalmol. Vis. Sci..

[16] Sullivan-Mee M, Katiyar S, Pensyl D, Halverson KD, Qualls C (2012). Relative importance of factors affecting corneal hysteresis measurement. Optom. Vis. Sci..

[17] Zhang B (2019). Associations with corneal hysteresis in a population cohort: Results from 96 010 UK Biobank participants. Ophthalmology.

[18] Suzuki Y, Kiyosawa M (2022). Cardiac hypertrophy may be a risk factor for the development and severity of glaucoma. Biomedicines.

[19] Medeiros FA (2013). Corneal hysteresis as a risk factor for glaucoma progression: A prospective longitudinal study. Ophthalmology.

[20] Bae SS, Menninga I, Hoshino R, Humphreys C, Chan CC (2018). Nomogram to predict graft thickness in Descemet stripping automated endothelial keratoplasty: An eye bank study. Cornea.

[21] Nahum Y, Leon P, Busin M (2015). Postoperative graft thickness obtained with single-pass microkeratome-assisted ultrathin Descemet stripping automated endothelial keratoplasty. Cornea.

[22] Sikder S, Nordgren RN, Neravetla SR, Moshirfar M (2011). Ultra-thin donor tissue preparation for endothelial keratoplasty with a double-pass microkeratome. Am. J. Ophthalmol..

[23] Busin M, Patel AK, Scorcia V, Ponzin D (2012). Microkeratome-assisted preparation of ultrathin grafts for descemet stripping automated endothelial keratoplasty. Investig. Ophthalmol. Vis. Sci..

[24] Romano V (2017). Preparation of ultrathin grafts for Descemet-stripping endothelial keratoplasty with a single microkeratome pass. J. Cataract Refract. Surg..

[25] Ruzza A (2021). Ultra-thin DSAEK using an innovative artificial anterior chamber pressuriser: A proof-of-concept study. Graefes Arch. Clin. Exp. Ophthalmol..

[26] Cheung AY (2018). Technique for preparing ultrathin and nanothin Descemet stripping automated endothelial keratoplasty tissue. Cornea.

[27] Kobayashi A, Yokogawa H, Mori N, Sugiyama K (2016). Visualization of precut DSAEK and pre-stripped DMEK donor corneas by intraoperative optical coherence tomography using the RESCAN 700. BMC Ophthalmol..

[28] Spadea L, Cerrone L, Necozione S, Balestrazzi E (2002). Flap measurements with the Hansatome microkeratome. J. Refract. Surg..

[29] Solomon KD (2004). Flap thickness accuracy: Comparison of 6 microkeratome models. J. Cataract Refract. Surg..

[30] Bates D, Mächler M, Bolker B, Walker S (2015). Fitting linear mixed-effects models Usinglme4. J. Stat. Softw..

