# Supplementary material for: The predictability of graft thickness for Descemet’s stripping automated endothelial keratoplasty using a mechanical microkeratome system
Source: Sci Rep. 2022 Dec 23;12:22210. doi: 10.1038/s41598-022-26679-w (PMC9789079; doi:10.1038/s41598-022-26679-w)
Supplement: Supplementary file 1 — Supplementary Figure 1. [file 41598_2022_26679_MOESM1_ESM.pdf]

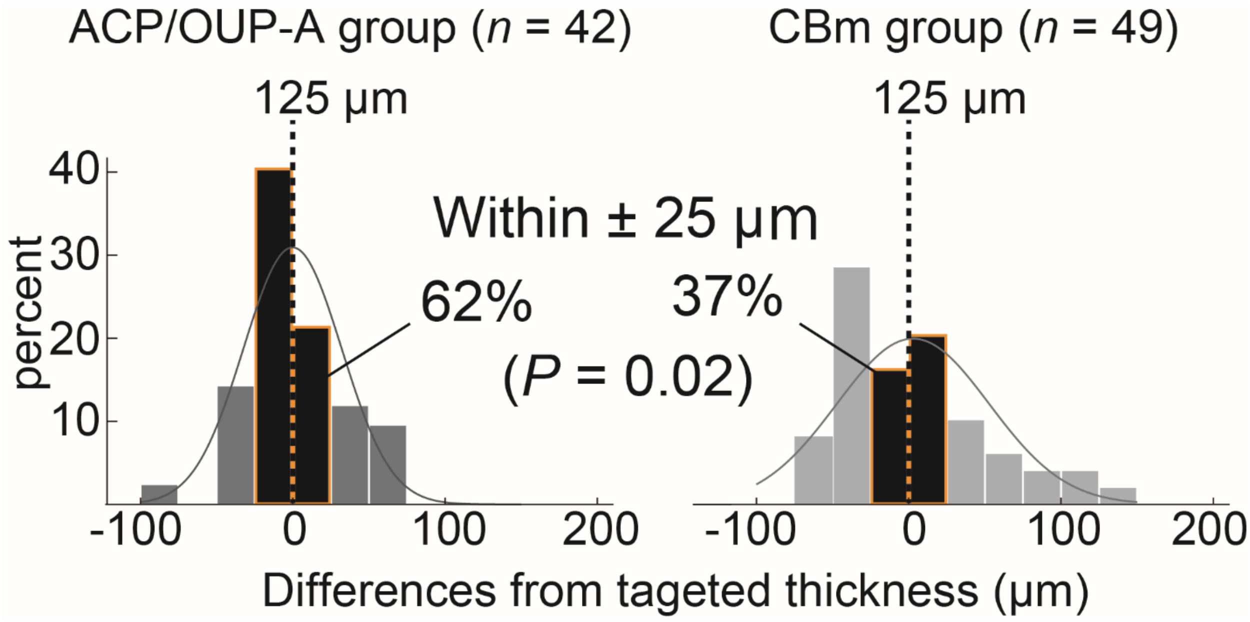


**Supplemental figure 1. Frequency distribution of the prepared DSAEK graft by two types of microkeratome groups cut mechanically or manually.** The donor tissue has been dissected by the mechanical microkeratome system using an artificial chamber pressurizer (ACP, Moria, Antony, France) and one use-plus automated (OUP-A, Moria, ACP/OUP-A group) or conventional method using an infusion bottle and Carriazo-Barraquer microkeratome with manual rotative cutting (CBm, Moria, CBm group). The donors with a corneal thickness of 490 µm to 570 µm, cut with a 350-head size are displayed. DSAEK grafts cut by ACP/OUP-A display a narrower thickness range and have been cut with higher accuracy than those obtained from CBm cutting (the range within ± 25µm, ACP/OUP-A group 62% *vs*. CBm 37%, $P = 0.02$). Differences between the target central graft thickness (125 µm, dashed line) and the actual measured thickness value are displayed. The solid line represents a normal estimate.
